# Cohort size required for prognostic genes analysis of stage II/III esophageal squamous cell carcinoma

**DOI:** 10.3389/pore.2023.1610909

**Published:** 2023-02-07

**Authors:** Linghong Kong, Ming Yang, Zhiyi Wan, Lining Wang

**Affiliations:** ^1^ Department of Pathology, Beijing Chuiyangliu Hospital, Beijing, China; ^2^ Hepato-Pancreato-Biliary Center, Beijing Tsinghua Changgung Hospital, School of Clinical Medicine, Tsinghua University, Beijing, China

**Keywords:** esophageal squamous cell carcinoma, prognostic genes analysis, power-law, events number, cohort size

## Abstract

**Background:** Few overlaps between prognostic biomarkers are observed among different independently performed genomic studies of esophageal squamous cell carcinoma (ESCC). One of the reasons for this is the insufficient cohort size. How many cases are needed to prognostic genes analysis in ESCC?

**Methods:** Here, based on 387 stage II/III ESCC cases analyzed by whole-genome sequencing from one single center, effects of cohort size on prognostic genes analysis were investigated. Prognostic genes analysis was performed in 100 replicates at each cohort size level using a random resampling method.

**Results:** The number of prognostic genes followed a power-law increase with cohort size in ESCC patients with stage II and stage III, with exponents of 2.27 and 2.25, respectively. Power-law curves with increasing events number were also observed in stage II and III ESCC, respectively, and they almost overlapped. The probability of obtaining statistically significant prognostic genes shows a logistic cumulative distribution function with respect to cohort size. To achieve a 100% probability of obtaining statistically significant prognostic genes, the minimum cohort sizes required in stage II and III ESCC were approximately 95 and 60, respectively, corresponding to a number of outcome events of 33 and 36, respectively.

**Conclusion:** In summary, the number of prognostic genes follows a power-law growth with the cohort size or events number in ESCC. The minimum events number required to achieve a 100% probability of obtaining a statistically significant prognostic gene is approximately 35.

## Background

Esophageal squamous cell carcinoma (ESCC) is the most common histologic subtype of esophageal cancer and characterized by a high degree of clinical and genetic heterogeneity [1–3]. A reliable set of prognostic genes will contribute to a better understanding of the molecular mechanisms of ESCC progression and is crucial to guide clinical management. With the development of high-throughput sequencing technology, whole-exome sequencing (WES) or whole-genome sequencing (WGS) has been widely used for prognostic markers analysis in ESCC [4–11]. Over 1,000 ESCC exomes have been sequenced in the past years, however, little overlap between prognostic genes has been seen in the different ESCC studies [4–11]. The most straightforward explanation for this phenomenon is usually attributed to the fact that the cohorts used in different studies differed in certain potentially relevant factors (such as stage, gender, and genetic context). However, sample size is also an important influencing factor [12].

Somatic mutations have been detected in the coding regions of approximately 14,000 genes in ESCC, of which 65 genes showed mutation frequency of >5% [8]. In prognostic survival analysis, the number of outcome events should be sufficient relative to predictors [13]. For the identification of prognosis-related genes, the cohort size should be larger than the number of mutated genes. However, the cohort size for different genomic studies in ESCC is usually in the tens to hundreds. In addition, the number of outcome events is also an important factor for prognostic genes analysis. The statistical power of survival analysis actually depends on the number of outcome events rather than the total cohort size [14, 15].

Here, our aim is to define how many cases are needed to identify prognosis-related genes in ESCC? To exclude other influencing factors, such as genetic context, follow-up time, and staging, we have focused here on a single ESCC dataset from one center and investigated the effect of cohort size on prognostic genes using random resampling methods.

## Methods

### Study data

Somatic mutation data and clinical information of ESCC cases were obtained from the published study [8]. Of the patients, a total of 222 ESCC patients with stage II and 165 ESCC patients with stage III from one center had overall survival (OS) data and were selected for further analysis. The median follow-up time for stage II and III patients was 34 and 27 months, respectively. The number of OS outcome events for stage II and III patients was 77 and 99, respectively.

### Prognostic genes analysis

Random resampling was performed by randomly selecting n cases in the dataset of stage II patients and the dataset of stage III patients, respectively. The cohort size n ranged from 1/7%–95% of all cases, with each cohort size being randomly sampled 100 times. Predictive analyses for prognostic genes associated with OS were also repeatedly performed 100 times for each cohort size level using the “maftools” package [16]. Survival analyses were determined using the Kaplan-Meier method and compared by the log-rank test. Differential genes with *p* < 0.05 were considered significant.

### Statistical analysis

Statistical analysis was carried out using SPSS 22.0 software (SPSS, Inc., Chicago, IL, United States) or R statistical software (v4.1.0; R Core Team 2021). All results were presented as means ± standard error.

## Results

**FIGURE 1:**
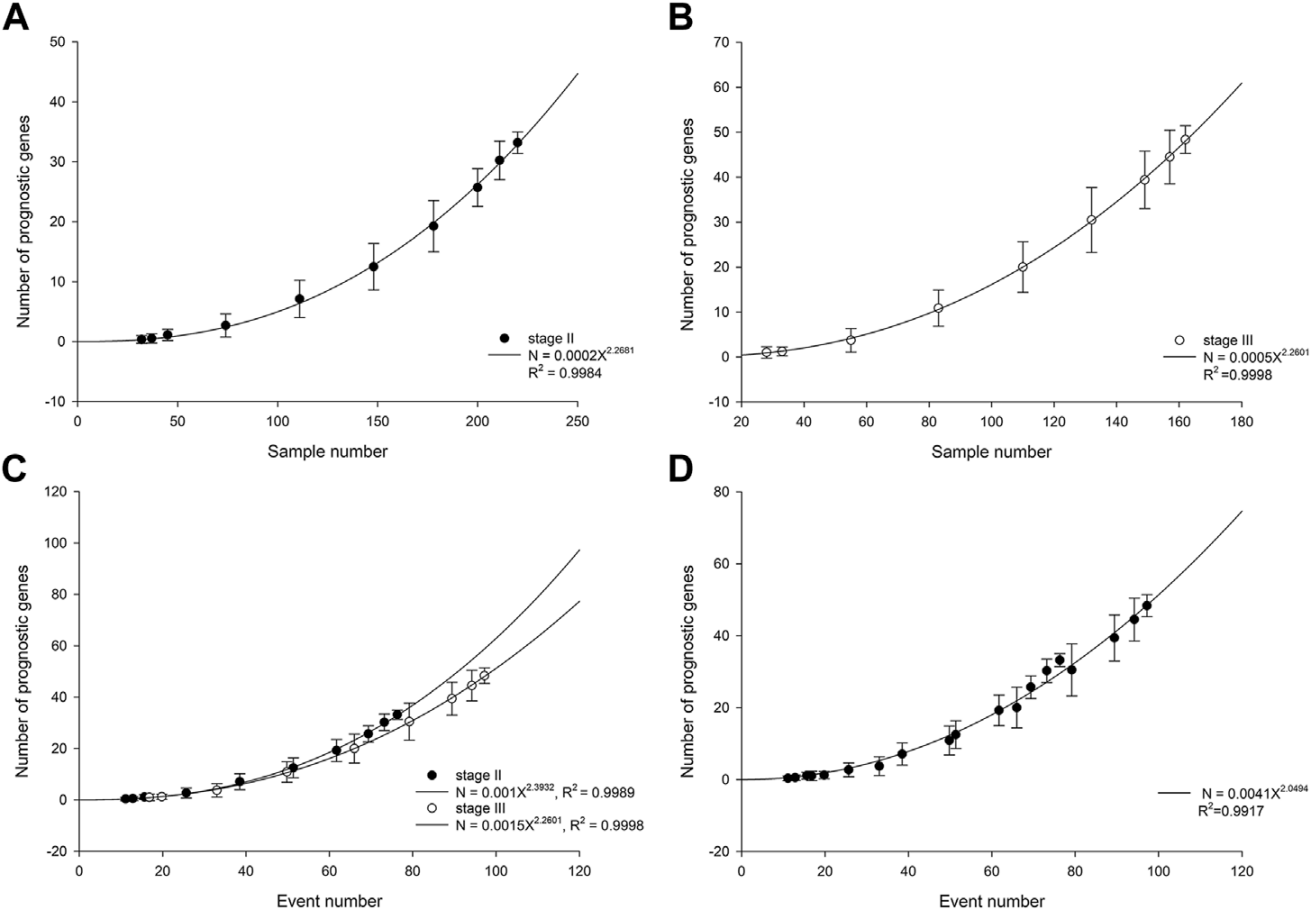
Number of prognostic genes increases with cohort size in stage II ESCC **(A)** and stage III ESCC **(B)**. The lines are best-fit results for power-law growth N = 0.0002X^2.2681^ in stage II ESCC and N = 0.0005X^2.2601^ in stage III ESCC, respectively. **(C)** Number of prognostic genes increases with events number. The lines are best-fit results for power-law growth N = 0.001X^2.3932^ in stage II ESCC and N = 0.0015X^2.2601^ in stage III ESCC, respectively. **(D)** Mixing data from stage II and stage III, simulated number of prognostic genes increases with events number. The line is best-fit result for power-law growth N = 0.0041X^2.0494^.

In patients with stage II ESCC, the number of statistically significant prognostic genes increased with cohort size in a power-law with an exponent of 2.27 (Figure 1A). The power-law growth curve was also observed in patients with stage III ESCC and was generally consistent with the growth exponent of patients with stage II ESCC (Figure 1B). We further analyzed the relationship between the number of prognostic genes and the number of outcome events. Power-law growth curves were observed again for stage II and stage III ESCC, respectively, and they almost overlapped (Figure 1C). We then simulated and analyzed the relationship between the number of prognostic genes and the events number by mixing data from stage II and stage III ESCC. The best-fit curve also conformed to the power-law growth with *R* ^2^ of 0.9917 (Figure 1D).

**FIGURE 2:**
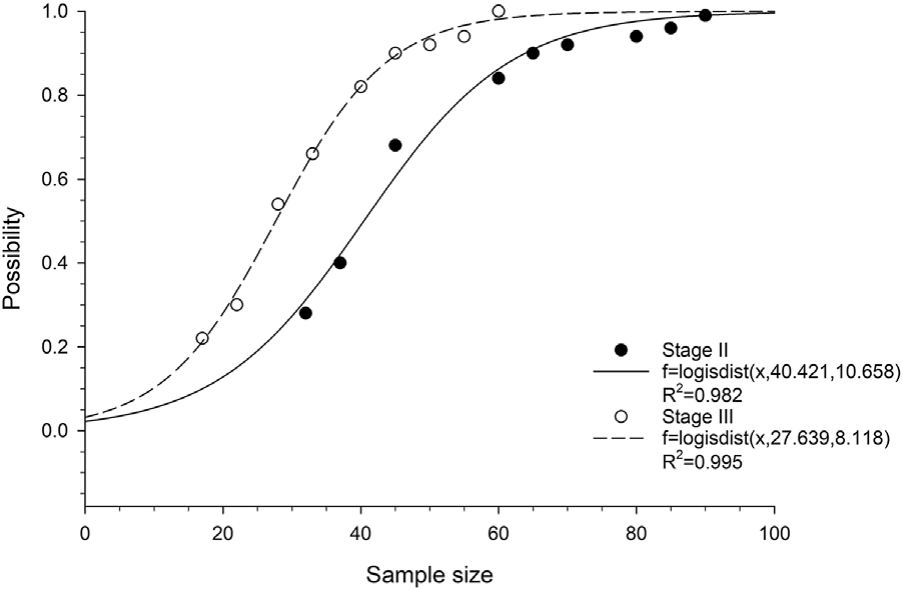
Correlation between probability of obtaining at least one prognostic gene and cohort size in ESCC patients. The fitted curves are based on logistic cumulative distribution functions.

We then analyzed the probability of obtaining at least one statistically significant prognostic gene in relation to cohort size. Logistic cumulative distribution curves were observed in both stage II and stage III ESCC patients (Figure 2). To achieve a 90% probability of obtaining statistically at least one statistically significant prognostic gene, the minimum cohort sizes required for stage II and III ESCC were approximately 65 and 45, respectively (Figure 2), which correspond to a number of events of 23 and 27, respectively. To achieve a 100% probability of obtaining at least one statistically significant prognostic gene, the minimum cohort sizes required for stage II and III ESCC were approximately 95 and 60, respectively (Figure 2), which correspond to a number of events of 33 and 36, respectively.

## Discussion

Prognosis is one of the core principles of medical practice. A number of studies have been conducted on the prediction of OS in ESCC based on WGS or WES [4–11]. However, few studies investigate the cohort size needed for prognostic genes studies. The prognostic genes identified by different studies showed very little overlap [4–11]. One reason is the inadequate cohort size. In this paper, we focused on a single ESCC dataset from one center and investigated the effect of cohort size on prognostic genes using random resampling methods. This cohort is the largest ESCC cohort to date from a single clinical center and includes 437 ESCC cases from Han population of Shanxi, China [8]. This cohort nicely excludes the interference of genetic background differences. A total of 387 patients with stage II/III ESCC were enrolled in the study after verification of clinical information.

In both stage II and stage III ESCC patients, the number of prognostic genes showed a power-law relationship with increasing cohort size, although the specific parameters of the formula differed. However, prognostic analysis is based on time-to-event data [17]. The events number is more critical than cohort size in prognostic analysis. Relative to the events number, the growth curves of the number of prognostic genes in patients with stage II and stage III ESCC largely overlapped. Although the cohort sizes required for prognostic genes analysis of stage II and stage III ESCC are different, the number of outcome events required is essentially the same. These results indicated that the power-law growth of the number of prognostic genes with cohort size is common in ESCC, independent of stage.

Prognostic studies are usually retrospective and cohort sizes are rarely considered prior to analysis [18]. However, the prognostic genes obtained on limited data are nothing but misleading. Our results showed that at least 35 outcome events are required in ESCC to ensure the acquisition of statistically significant prognostic genes.

A limitation of this study is that the number of ESCC patients from one center (99 outcome events) is still insufficient, resulting in the plateau in the number of prognostic genes not reached. Enrolling more patients will detect more mutated genes, thereby increasing the number of prognostic genes. However, theoretically there should be a plateau in the number of prognostic genes. The number of cases or events needed to reach this plateau still needs to be further explored.

## Conclusion

In summary, the number of prognostic genes takes a power-law growth with cohort size in ESCC. Our results suggest that at least 35 outcome events are required for genomic mutation-based prognostic studies in ESCC. These results will help to the trial design of prognostic genes analysis in ESCC.

## Data Availability

The original contributions presented in the study are included in the article/supplementary material, further inquiries can be directed to the corresponding author.

## References

[1] Lam AK. Molecular biology of esophageal squamous cell carcinoma. Crit Rev Oncol Hematol (2000) 33:71–90. 10.1016/s1040-8428(99)00054-2 10737369

[2] Arnal MJD Arenas ÁF Arbeloa ÁL. Esophageal cancer: Risk factors, screening and endoscopic treatment in Western and Eastern countries. World J Gastroenterol (2015) 21:7933–43. 10.3748/wjg.v21.i26.7933 26185366 PMC4499337

[3] Lin DC Wang MR Koeffler HP. Genomic and epigenomic aberrations in esophageal squamous cell carcinoma and implications for patients. Gastroenterology (2018) 154:374–89. 10.1053/j.gastro.2017.06.066 28757263 PMC5951382

[4] Lin DC Hao JJ Nagata Y Xu L Shang L Meng X Genomic and molecular characterization of esophageal squamous cell carcinoma. Nat Genet (2014) 46:467–73. 10.1038/ng.2935 24686850 PMC4070589

[5] Gao YB Chen ZL Li JG Hu XD Shi XJ Sun ZM Genetic landscape of esophageal squamous cell carcinoma. Nat Genet (2014) 46:1097–102. 10.1038/ng.3076 25151357

[6] The Cancer Genome Atlas Research Network. Integrated genomic characterization of oesophageal carcinoma. Nature (2017) 541:169–75. 10.1038/nature20805 28052061 PMC5651175

[7] Moody S Senkin S Islam SMA Wang J Nasrollahzadeh D Cortez Cardoso Penha R Mutational signatures in esophageal squamous cell carcinoma from eight countries with varying incidence. Nat Genet (2021) 53:1553–63. 10.1038/s41588-021-00928-6 34663923

[8] Cui Y Chen H Xi R Cui H Zhao Y Xu E Whole-genome sequencing of 508 patients identifies key molecular features associated with poor prognosis in esophageal squamous cell carcinoma. Cell Res (2020) 30:902–13. 10.1038/s41422-020-0333-6 32398863 PMC7608103

[9] Song Y Li L Ou Y Gao Z Li E Li X Identification of genomic alterations in oesophageal squamous cell cancer. Nature (2014) 509:91–5. 10.1038/nature13176 24670651

[10] Sawada G Niida A Uchi R Hirata H Shimamura T Suzuki Y Genomic landscape of esophageal squamous cell carcinoma in a Japanese population. Gastroenterology (2016) 150:1171–82. 10.1053/j.gastro.2016.01.035 26873401

[11] Zhang N Shi J Shi X Chen W Liu J. Mutational characterization and potential prognostic biomarkers of Chinese patients with esophageal squamous cell carcinoma. Onco Targets Ther (2020) 13:12797–809. 10.2147/OTT.S275688 33363385 PMC7751839

[12] Ein-Dor L Kela I Getz G Givol D Domany E. Outcome signature genes in breast cancer: Is there a unique set? Bioinformatics (2005) 21:171–8. 10.1093/bioinformatics/bth469 15308542

[13] Riley RD Snell KI Ensor J Burke DL Harrell FE Jr Moons KG Minimum sample size for developing a multivariable prediction model: PART II - binary and time-to-event outcomes. Stat Med (2019) 38:1276–96. 10.1002/sim.7992 30357870 PMC6519266

[14] Schober P Vetter TR. Survival analysis and interpretation of time-to-event data: The tortoise and the hare. Anesth Analg (2018) 127:792–8. 10.1213/ANE.0000000000003653 30015653 PMC6110618

[15] In J Lee DK. Survival analysis: Part II - applied clinical data analysis. Korean J Anesthesiol (2019) 72:441–57. 10.4097/kja.19183 31096731 PMC6781220

[16] Mayakonda A Lin DC Assenov Y Plass C Koeffler HP. Maftools: Efficient and comprehensive analysis of somatic variants in cancer. Genome Res (2018) 28:1747–56. 10.1101/gr.239244.118 30341162 PMC6211645

[17] Moons KG Royston P Vergouwe Y Grobbee DE Altman DG. Prognosis and prognostic research: What, why, and how? Bmj (2009) 338:b375. 10.1136/bmj.b375 19237405

[18] Jinks RC Royston P Parmar MK. Discrimination-based sample size calculations for multivariable prognostic models for time-to-event data. BMC Med Res Methodol (2015) 15:82. 10.1186/s12874-015-0078-y 26459415 PMC4603804

